# Proposed solutions to anthropogenic climate change: A systematic literature review and a new way forward

**DOI:** 10.1016/j.heliyon.2023.e20544

**Published:** 2023-10-10

**Authors:** Svetlana V. Feigin, David O. Wiebers, George Lueddeke, Serge Morand, Kelley Lee, Andrew Knight, Michael Brainin, Valery L. Feigin, Amanda Whitfort, James Marcum, Todd K. Shackelford, Lee F. Skerratt, Andrea S. Winkler

**Affiliations:** a All Life Institute, Washington, D.C., USA; b Mayo Clinic and Mayo Foundation, Rochester, MN, USA; c Centre for the Study of Resilience and Future Africa, University of Pretoria, Pretoria, South Africa; d Ministry of Environment, Forest and Climate Change (MoEFCC), India; e Faculty of Veterinary Technology (CNRS), Kasetsart University, Bangkok, Thailand; f Faculty of Tropical Medicine, Mahidol University, Bangkok, Thailand; g Pacific Institute on Pathogens, Pandemics and Society, Faculty of Health Sciences, Simon Fraser University, Burnaby, British Columbia, Canada; h Global Health Governance, Canada; i School of Environment and Science, Nathan Campus, Griffith University, Nathan, QLD, Australia; j Faculty of Health and Wellbeing, University of Winchester, Winchester, UK; k Clinical Neurosciences and Preventive Medicine, Danube University Krems, Austria; l National Institute for Stroke and Applied Neurosciences, School of Clinical Sciences, Auckland University of Technology, New Zealand; m Department of Professional Legal Education, Faculty of Law, The University of Hong Kong, Hong Kong; n Department of Philosophy, Baylor University, Waco, TX, USA; o Department of Psychology and Center for Evolutionary Psychological Science, Oakland University, Rochester, MI, USA; p Melbourne Veterinary School, Faculty of Science, University of Melbourne, Melbourne, Victoria, Australia; q Center for Global Health, Department of Neurology, Faculty of Medicine, Technical University of Munich, Munich, Germany; r Department of Community Medicine and Global Health, Institute of Health and Society, Faculty of Medicine, University of Oslo, Norway

## Abstract

Humanity is now facing what may be the biggest challenge to its existence: irreversible climate change brought about by human activity. Our planet is in a state of emergency, and we only have a short window of time (7–8 years) to enact meaningful change. The goal of this systematic literature review is to summarize the peer-reviewed literature on proposed solutions to climate change in the last 20 years (2002–2022), and to propose a framework for a unified approach to solving this climate change crisis. Solutions reviewed include a transition toward use of renewable energy resources, reduced energy consumption, rethinking the global transport sector, and nature-based solutions. This review highlights one of the most important but overlooked pieces in the puzzle of solving the climate change problem – the gradual shift to a plant-based diet and global phaseout of factory (industrialized animal) farming, the most damaging and prolific form of animal agriculture. The gradual global phaseout of industrialized animal farming can be achieved by increasingly replacing animal meat and other animal products with plant-based products, ending government subsidies for animal-based meat, dairy, and eggs, and initiating taxes on such products. Failure to act will ultimately result in a scenario of irreversible climate change with widespread famine and disease, global devastation, climate refugees, and warfare. We therefore suggest an “All Life” approach, invoking the interconnectedness of all life forms on our planet. The logistics for achieving this include a global standardization of Environmental, Social, and Governance (ESG) or similar measures and the introduction of a regulatory body for verification of such measures. These approaches will help deliver environmental and sustainability benefits for our planet far beyond an immediate reduction in global warming.

## 1 Introduction

### 1.1 The problem

Life on our planet is considered to be undergoing a sixth mass extinction brought about primarily as a result of human activity [[1], [2], [3], [4], [5]]. Since industrialization began in the mid-18th century, our exploitation of natural resources (e.g., water, land, fossil fuels) as though they are infinite, pollution of the environment, and reliance on animal agriculture, have resulted in unprecedented global changes to biodiversity, ecosystems, global pandemics, and the Earth's climate [3,4,[6], [7], [8]]. Although there are numerous environmental threats such as biodiversity loss and pollution, climate change is the single gravest immediate threat facing humanity [9,10], and we are in a state of planetary emergency [11]. Any solutions to climate change should include other environmental threats (such as biodiversity loss) and be guided by the principle of interconnectedness of all life forms on our planet [4,6,7,12,13]. Due to the anthropogenic release of greenhouse gases, a global average temperature increase of more than 1 °C has already occurred (relative to pre-industrial times) [8]. The human and planetary consequences of this increase already can be seen with severe climate events, displacement of humans and animals due to climate catastrophes, failing crops and starvation, and increased risk of pandemics [[14], [15], [16], [17], [18]]. Scientists estimate that we have a 7–8-year time window to enact substantial changes before the effects of climate change are irreversible [3,11].

It is now widely accepted among scientists that far-reaching global change in human behavior is needed if we are to evade potentially irreversible climate change [19]. Specifically, if we are to limit global warming to 1.5 °C above pre-industrial levels, global annual greenhouse gas emissions need to be reduced by 45% by 2030 (at the present trajectory, they are projected to rise by 10–15%) [2,3,11]. Warming beyond 1.5 °C will put us on a course of irreversible damage and a climate crisis [2,3,9,11,20]. The choices we make, from governments to corporate entities and individual consumers, are now of the utmost importance and have global consequences for the planet and all life on it. Humanity is facing one of its most important challenges, and as will become clear in this literature review, the requisite changes in human behavior must be rapid and global and include dietary changes, changes in our reliance on fossil fuels, and international cooperation in achieving climate change mitigation. A gradual global shift to a plant-based diet and the gradual global phaseout of industrialized animal agriculture also reduce the risk of zoonoses and future pandemics and should be part of an integrated global pandemic preparedness response [[21], [22], [23], [24], [25]].

This literature review summarizes research advocating for the mitigation of climate change through a reduction in energy use and energy substitution, changes to the transportation sector, ending of deforestation, and changes to agriculture and human consumption, and, in the context of such research, discusses the path forward. Specifically, in Section 2 (Methods), the authors describe how the literature search was conducted and in Section 3 (Results), the authors present all proposed solutions to climate change in the last 20 years (2002–2022). The solutions are grouped according to their proposed mitigation strategy (e.g., transition toward renewable energy sources). Section 4 (Discussion) includes a proposal of three strategic approaches for mitigating climate change, and a Conclusion follows in Section 5.

## 2 Methods

**Fig. 1:**
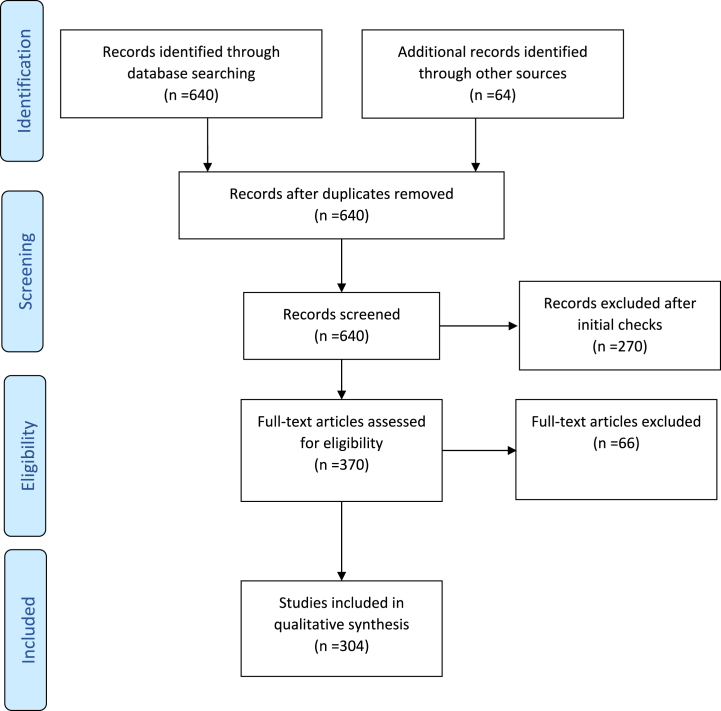
Flowchart of literature search showing the number of records identified, excluded, and included. Adapted from Moher D, Liberati A, Tetzlaff J, Altman DG. Preferred reporting items for systematic reviews and meta-analyses: the PRISMA statement. BMJ: British Medical Journal. 2009; 339 (7716):332–6.

A systematic literature review was conducted using PRISMA (Preferred Reporting Items for Systematic Reviews and Meta-Analyses) criteria as a guideline [26] to summarize the peer-reviewed literature on proposed global solutions to climate change in the last 20 years (2002–2022) (Fig. 1). It was surmised that articles older than 20 years may present out-of-date science and any relevant mitigation strategies proposed prior to 2002 are likely to be mentioned within the timeframe of this review if they were still relevant today. Research literature was sourced from several major databases between October and December of 2022 (ScienceDirect, GreenFile, Google Scholar, Scopus, JSTOR, PsycINFO, SAGE journals, SpringerLink) as well as secondary sources such as reference lists from accessed articles (Table 1). A total of 704 publications were retrieved (prior to duplicate removal). Keywords used in the search were solutions, mitigation, strategies, prevention, climate change, and global warming. Duplicates were removed (*n* = 64).

**Table 1:** Databases searched (prior to duplicate removal) (n = 704).

Databases searched (prior to duplicate removal)	Number of records
ScienceDirect	65
GreenFile	35
Google Scholar	130
Scopus	270
JSTOR	21
PsycINFO	25
SAGE journals	17
Springerlink	77
Hand search	64

Sourced literature was initially screened and assessed for eligibility using a pre-determined eligibility checklist (Table 2a). Initial screening involved scanning all sourced publications for relevance to the topic of proposed global solutions to climate change according to keywords, title, and abstract. Direct mention had to be made of proposed solutions to climate change in the keywords, title or abstract. Mention of proposed solutions or strategies to climate change or global warming (without explicitly stating ‘human-caused’) and mitigation (or prevention) of climate change also was accepted, provided it was relevant (e.g., if the presentation of solutions to climate change was the main objective of the article). Global solutions to climate change refers to proposed solutions in a global context (such as the switch to renewable energy). Studies which did not pass initial screening were excluded. In total, 270 items were excluded during the initial screening (Table 3).

**Table 2a:** Eligibility checklist used for initial screening (title, abstract, keywords).

Checklist
Direct mention of solutions to climate change in keywords and/or title and/or abstract; solutions to climate change can also be referred to as mitigation and/or mitigation strategies
Climate change referred to as global warming accepted
Mitigation referred to as prevention if in the context of climate change
Peer-reviewed article

**Table 2b:** Eligibility checklist used for full-text check (after article passed initial screening).

Checklist
Solutions to climate change as main topics in body of article; solutions to global warming; mitigation strategies; prevention;
Climate change referred to as anthropogenic or human caused
Peer reviewed
English and full-text

**Table 3:** A breakdown of records excluded after initial and full-text checks (n = 336) with reasons.

Reason for exclusion	Number of records
Theology/media/thesis	8
Climate change perceptions without solutions	24
Adaptation/impact without solutions/mitigation	28
Book review/book	12
Not meeting criteria (abstract, keywords)	161
Unavailable (e.g., not English)	37
Not relevant (after full text review)	66

Following initial screening, full-text eligibility checks were conducted on all remaining records (*n* = 370). In accordance with PRISMA recommendations [26], a full-text eligibility checklist was devised to assist with the final screening of publications (Table 2b). A total of 66 items were removed during full-text eligibility checks (Table 3). All items that passed eligibility were then critically reviewed and synthesized (a total of 304 items).

## 3 Results

### 3.1 General considerations

There are two broad approaches to the climate change crisis – adaptation and mitigation. Adaptation refers to responding to the effects of climate change in both precautionary and reactive ways rather than through the preventive approach of mitigation [27]. Mitigation refers to reducing the sources or increasing sinks of greenhouse gas (GHG) emissions. Mitigation policies (also referred to as abatement) and their effectiveness are affected by two factors. The first is climate inertia, which refers to the period of time it takes the climate to reach equilibrium after GHG stabilization [28,29]. The second limit on mitigation's effectiveness is the number of countries that commit to GHG emission reductions, commitments that often lack stringency [28].

Proponents of adaptation over mitigation strategies argue that we can develop technology/lifestyles to cope with a climate-changed world [30]. This argument fails to acknowledge the devastating impact climate change has and will have on low- and middle-income-countries (LMICs). This argument also fails to consider the effects that irreversible changes will have on the planet, to which adaptation may prove difficult, if not impossible. Further, more environmental damage is done when countries invest in adaptation measures before mitigation measures [28]. This increased damage occurs because countries that adopt adaptation approaches take advantage of a strategic effect and, in turn, invest less in mitigation, which does little to help solve the climate change crisis [28]. The most effective approach combines adaptive and mitigating measures simultaneously [28].

The present literature review synthesizes proposed solutions (mitigation strategies) to anthropogenic climate change. Although the proposed solutions discussed in this review are categorized, no single solution on its own is sufficient for mitigating the climate crisis. Such an approach can lead to overestimating or underestimating the potential of solutions to reduce carbon [31].

### 3.2 Proposed solutions

#### 3.2.1 Transition toward renewable energy sources

Humanity's heavy reliance on and consumption of fossil fuels has resulted in the increasing of the atmospheric concentration of greenhouse gases, particularly Carbon Dioxide (CO₂) [[32], [33], [34]]. CO₂ accounts for 78% of total GHG emissions [33], and 68% of global GHG emissions is attributable to fossil fuels [35]. Two solutions have been proposed to address the growing emissions of CO₂ from anthropogenic sources: a reduction of energy use by way of improved energy efficiency and transitioning to low/zero carbon energy sources, such as renewables [32,[36], [37], [38], [39], [40], [41], [42]]. Researchers examining which of the two strategies (i.e. energy efficiency and renewable energy) is most effective in curbing CO₂ emissions have found that renewable energy has a slight advantage over energy efficiency [32]. Authors of this study point out, however, that curbing unnecessary energy demands needs to go together with both strategies [32].

Examples of renewable energy sources include hydropower, fuelwood, biomass, vegetable oils, biogas, geothermal energy, solar heating/cooling of buildings, tidal and thermal ocean energy, solar drying [41,[43], [44], [45], [46], [47]], and clean technology adoption within the minerals sector (i.e. cement, glass, ceramic, lime) [48]. For example, the burning of biomass (e.g. wood pellets) for energy, as long as this biofuel is sustainably sourced, has the potential to form part of the solution portfolio to climate change [49]. Transitioning to renewable energy sources involves developing and widely implementing affordable renewable energy sources (e.g., wind and solar power) [32,44,50,51]. If countries are to shift to reliance on renewable energy sources (e.g., solar, hydraulic, wind, geothermal, biomass), other energy producing technologies will need to be developed to keep the electricity network stable during periods of little to no renewable energy production [52]. Researchers have proposed several techniques to cope with the intermittent nature of renewable energy sources, including batteries and power-to-gas technologies (converting surplus renewable energy into hydrogen gas) [44,52,53]. Hydrogen gas can then be injected into the natural gas grid, thereby reducing GHG emissions and our reliance on high-carbon fuels [52,54,55].

Growing urbanization in LMICs (such as India) means increased demand for energy. Renewable energies have been suggested as potentially playing a role in ending energy poverty in LMICs [37]. Reducing black carbon emissions [56], by replacing coal and oil consumption with natural gas has been suggested for both LMICs [44] and worldwide [57], with high-emitting countries like China specifically urged to also adopt hydroelectric, wind, solar, nuclear, and biomass technologies [58]. Researchers have proposed a renewable energy grid integration in the design of ‘smart’ cities (environmentally-sustainable cities in LMICs) involving ‘smart’ metering and grid level storage [59]. Other renewable energy solutions include cogeneration (i.e. capture of waste heat), rooftop solar systems and solar farms, capturing landfill methane [31,41,60,61], capturing farm methane [45], ‘clean’ coal [62], carbon capture and storage (CCS) technology [44,45,63], hydrogen fusion technology, natural refrigerants [64], and nuclear energy [44,45,[65], [66], [67]].

Renewable energy solutions which are in their infancy or considered to be controversial include hydrogen fusion technology, nuclear energy, and utilization of CO₂ by converting it into fuels. Solutions such as hydrogen fusion technology will likely not become viable on a scale large enough and affordable enough for mass energy production for another 20–30 years [67]. Similarly, some scholars have proposed the utilization of CO₂ by thermochemical, photochemical, and electrochemical pathways to be converted into fuels, chemical feedstock, and concrete [68]. This approach, however, is in its infancy and assumes a 'business-*as*-usual' model for industrial development and urbanization [68]. Nuclear energy is a zero-carbon energy which can be integrated into existing infrastructure and provide for future energy needs [44,45,65]. Public concerns need to be addressed first, however, such as those of safety, management of waste, storage, and weaponization [44,45,69,70]. Also, some critics of nuclear energy argue that widespread use of nuclear power is too expensive and would take too long to achieve to be a viable climate solution, contending that the focus needs to be directed at energy efficiency and renewable energy instead [69]. Another example of an ambitious proposal is to use large sailing ships to create storable energy (hydrogen) by converting ocean wind power [71,72]. However, such an approach is logistically challenging as a means of meeting global energy demands [71].

Some scholars argue that solutions to climate change mitigation already exist and it is a matter of scaling them up to achieve climate goals [73]. For example, to stabilize the climate and solve the problem for the next half-century, technologies such as biofuels, wind electricity, renewable hydrogen, soil management, and photovoltaic electricity can be scaled up and used in combination [73]. Turning to renewable energy, however, may increase reliance on other resources (e.g., wood, metals) and neglect other mitigation strategies (such as lifestyle changes) which, in turn, may negatively impact the environment such as loss of biodiversity and land use change [32,49,74,75]. For example, increased exploitation of biofuels may lead to further deforestation and monoculture plantations, in turn, increasing biodiversity loss [49]. Also, mining for materials such as metals used in renewable energy production can threaten biodiversity by impinging on protected wilderness areas [75]. Furthermore, the push for renewable energy involves substantial initial investment, requiring large amounts of energy which would come predominantly from fossil fuels [76]. Meeting projected global demands for energy with only renewable energy sources will likely take too much time and not occur at the pace required by the targets of the Paris Agreement [76].

#### 3.2.2 Reducing the consumption of energy, energy efficiency improvement, and moving beyond a consumer culture

Scholars have argued that for technological solutions to be successful in addressing climate change, they must be accompanied by reductions in consumption and production [[76], [77], [78], [79], [80]]. Although a carbon tax would be one way to reduce consumption, public support for such a measure is low [81]. Reducing energy consumption involves conservation efforts coupled with behavioral changes (such as increasing beliefs about individual and collective efficacy) [82], and energy efficiency improvement involving technological innovations [32].

Reducing emissions from decreased use of electricity can be achieved through demand side energy efficiency solutions [45]. For example, numerous technologies are available (such as fluorescent lighting, low water using devices) that reduce energy consumption while meeting industrial, residential, and commercial energy demands (e.g., heating, lighting, and refrigeration) [45]. Improving energy efficiency to reduce energy consumption can, however, have a rebound effect whereby energy efficiency increases consumption, for which this strategy has been criticized as a sole solution to the CO₂ problem [32].

To avoid the rebound effect, individuals must be intrinsically motivated (i.e. rather than being extrinsically motivated by, for example, financial incentives) to forego personal benefits in the form of commodities and convenience [80]. Intrinsic motivation to forego benefits such as convenience is challenging to achieve and research is lacking in this area [80]. Education initiatives, however, have been proposed as a starting point [80,83], as well as government incentives in the form of material support [84,85]. Moreover, there is a school of thought arguing against globalization and a re-localization of trade [78,86]. No state or country alone can solve the problem of climate change; it is the collective effort that can produce change [[87], [88], [89], [90], [91]]. Research also has suggested that successful mitigation requires both diverse city-level and national-level actions [[92], [93], [94]]. If democratic countries work collectively to mitigate the problem, then other countries may be more inclined to join the collective effort [95].

Any gains of technological innovations are offset by increased affluence and consumerism in society [36,76,80]. The reluctance of governments to promote changes in individual behavior may be explained by voter considerations or the lack of far-sightedness by voters and policymakers [77,95]. Further, proactive climate change mitigation policies may be perceived as threatening the economic growth of a country [36]. For example, in countries reliant on primary industries (such as New Zealand), protecting the economic interests of industries such as animal agriculture becomes intertwined with protecting ‘national interests’ [36].

Reduction in consumerism should be targeted at the affluent sectors of society. For example, a change in consumption habits in a high-income context with the highest mitigation potential includes a switch to renewable energy, adoption of a vegan (or less animal-based) diet and lifestyle, car-free or electric car transport, and less long-haul flying [40]. Support for this argument comes from research showing that income level is the primary predictor of energy use and GHG emissions. Specifically, the higher the income, the more energy is used and the higher the GHG emissions [[96], [97], [98]]. Conversely, strategies for reduced consumption in low-income countries may focus on providing basic necessities in the context of healthy consumption habits [76] such as advanced and ‘clean’ fuels for cooking, e.g., replacing solid fuel cooking (such as cow dung, coal, wood) with solar and biogas plants to reduce cooking emissions [[99], [100], [101]]. Governments need to support a shift towards sustainable/green commodities and should be cognizant of how income inequality can impact efforts toward transitioning to sustainable/green economies [102,103].

The global response to the climate crisis needs to be one of equity. LMICs with low emissions per capita should not have the same climate mitigation responsibilities as wealthy, industrialized countries [104] that have benefitted the most from climate change-inducing activities [105]. This includes the lowest income countries and small island nations that are disproportionately affected by anthropogenic climate change [105]. Each country's climate response depends on its cumulative, historical contribution to climate change, its current contribution, and its ability to respond [9,106]. Furthermore, reaching net-zero may not be an appropriate goal for collectivist action regarding climate change as differences exist as to how net-zero is defined [107]. The definition of net-zero needs to be standardized internationally by way of good governance, policy, and regulation [107]. The onus is, in effect, on wealthier countries to respond more quickly by significantly reducing emissions by 2030 [9,31,36,108] and supporting LMICs in their climate responses [9].

#### 3.2.3 Information technology and Artificial Intelligence as green solutions

Information Communication Technology Services (ICTs) account for about 2% of the ongoing anthropogenic environmental pollution [109]. The total electricity consumption of ICTs is expected to increase and recent data seems to suggest it has been on the rise [109,110]. Green ICTs and Green ISs (Information Systems) have been proposed within several areas; for example, using innovative energy use and saver systems, using renewable energy sources, re-cycling, re-using, reducing e-waste, and mobile/internet services to minimize required energy (e.g., teleconferencing, cloud computing, digital publication), zero power ICT solutions [55,108,109,111], and designing/implementing ISs that assist with achieving sustainability goals [55,112,113].

Green ICTs can be used to support the development and improvement of natural environment and resources surveillance systems to protect and restore natural ecosystems [109,111]. Also, big data analytics offer the possibility of precision agriculture and a more efficient use of resources such as water [111]. Annual improvements in Information Technologies (IT) also are conducive to energy use reduction [114]. For example, authors argue that an improvement in smartphone technology will lead to increased public transportation usage as it improves accessibility and information/location sharing, such as via GPS technology [114], and mass adoption of blockchain technologies can help reduce deforestation by creating a paperless world [115]. So far, however, digitalization has increased energy consumption and this increase has been greater than the ability of ICTs to reduce energy consumption [113].

The use of web-based social media and games have been suggested to educate the public on climate change and encourage sustainability and climate action on the part of individuals [112,116,117]. For example, the availability of Massive Open Online Courses, provides a focused space to generate discourse on climate change and potential solutions [116]. Also, Artificial Intelligence (AI) has been proposed as a tool to combat global climate change [111,118]. AI's potential benefits for addressing climate change include understanding and facilitating effective responses, particularly for complex tasks, informing policy-making [118], and facilitating environmental governance [119]. There are some limitations, however, of greenifying IT and AI, such as their dependence on electricity from non-renewable resources, carbon emissions, electronic waste, and the unsustainability of precious minerals which are used in hardware production [120].

#### 3.2.4 Global transport sector

The global transport sector is responsible for 19–26% of energy related GHG emissions [[121], [122], [123]]. Among the main contributors are road freight, car use, and aviation [121]. Road transport is responsible for 81% of GHG emissions within the transport sector, road freight being the largest emitter at 43% and motor cars the second largest [121]. Scientists have argued that across all impact categories, public transportation is more sustainable than private car use and have advocated for a modal shift to public transportation (e.g., metro and rail) as part of climate change mitigation [37,41,60,121,[124], [125], [126]]. Also, car sharing and demand management are suggested [126,127]. Other research has found that the highest reduction potential, in terms of private consumption habits, is flying less and using an electric vehicle (or living car-free) [40,128].

Other specific examples of GHG emission-reduction within the transportation sector include energy-efficient cars and trucks, alternative mobility such as walking and cycling [31,41,53,60,108,121,123,129], private car taxation [121], bicycle tourism [130], higher car occupancy [125], and sustainable mobility traffic policies (e.g., speed limit reduction, increased bike access) [126,129,131]. Alternative mobility, such as cycling, will require unprecedented behavioral change by individuals [123]; policy changes and investments will be necessary to make such alternative transport options accessible and attractive [121]. For example, researchers argue that mobility management campaigns need to focus on normalizing sustainable transport behavior and require national coordination [132]. Further, freight transportation reliance can be reduced by an increase in use of rail and sea freight [121,129]. The focus in high-income countries should be on the rate of transition from private to public modes of transportation because a shift to ‘greener’ private cars will not happen fast enough or widespread enough [125].

Public transportation, however, is not without environmental impacts, given its reliance on resources such as electricity and water. Researchers have proposed using cleaner renewable energy sources to mitigate these impacts (such as wind, solar power, hydrogen, and electric rail) [[52], [53], [54],^121^,^124^]. For example, hydrogen systems have lower GHG emissions when compared to other alternative fuels (e.g., hybrid vehicles, natural gas) [54,125,133]. Nuclear energy is used in some countries to power electrified transportation networks, such as France's rail system, which may exacerbate negative impacts of expanding public transportation including nuclear waste, accident risk, and increased biomass [133].

Decarbonizing land transportation in emerging economies has been identified as one of the major challenges of climate change mitigation [10,134], largely due to a growing demand in mobility and motorization in these economies. Reduction in CO₂ in emerging economies has been suggested through means of improvement of fuel economy (e.g., hybrid and electric vehicles) [53,135], improved public transportation [129], natural gas and hydrogen motors [121,125,134], and eliminating subsidies for oil products to promote alternate fuels [136] such as biofuels [[137], [138], [139], [140]]. Although some of these solutions offer promising directions, they will require advances in technology. For example, biofuels are already in use in some countries (such as bioethanol in India), however, technological advancement is needed to make biofuels an economically viable option for emerging economies [138,141].

The development of biofuels could greatly benefit the aviation industry, which accounts for approximately 2% of human-made CO₂ emissions and 5% of cumulative global warming [142]. Replacing fossil fuels with sustainable aviation fuels (e.g., biofuels) has been argued to be a potential short-term solution [138,143]. Other suggested steps in ‘greening’ the aviation industry include changing the business model [142], increasing fuel efficiency through upgraded equipment/procedures, upgraded aircraft, and optimizing routes and procedures [[143], [144], [145]]. Further suggestions are a reduction in short-haul flights by diverting passengers to inter-city rail travel, a reduction in air freight to transportation of perishable goods only [121], and weight reduction of aircraft using advanced (e.g., composite) materials to reduce fuel burn, reduction of drag, managing the aviation operational system more efficiently, and improving propulsive efficiency [145,146]. Overall, a rethinking and restructuring of the global transportation sector is necessary to reduce its impact on global climate change. Significant changes in public and private consumption and behaviors are necessary along with the decarbonization of land transport, and a switch to renewable fuels. Advances in technology, policy changes and national coordination, significant investment in alternative modes of transport, and normalization of sustainable transport behavior are necessary but present challenges in achieving climate change mitigation goals within the global transportation sector.

#### 3.2.5 Built environment and materials

Approximately 38% of global GHG emissions are attributable to the building sector (including building construction and material manufacturing) [147,148]. Waste management [149,150] including wastewater [151,152], recycling, renewable products, lower-carbon construction, more density housing [153,154], a reduction in per capita floor space [155,156], and building energy efficiency are thus important considerations for climate change mitigation within the building sector [39,73,157,158]. Examples of how to decarbonize and raise the building sector's energy efficiency include ultra-high performance cement composites, nanofibers instead of steel, sustainable wood materials [146,148], and geosynthetics [159]. However, an increased demand for forest products may have a detrimental effect on forest biodiversity, carbon sequestration rates (i.e. old growth forests sequester more carbon than monoculture forests) [160], and forest-dwelling communities [148]. Rammed earth (e.g., clay, sand, gravel) with a small amount of cement and water has also been suggested as an alternative building material for sustainable housing that has environmental and structural stability [161]. Strategies to decarbonize the appliances used in the building sector include refrigerant management, reduction of hydrofluorocarbon (HFC) and chlorofluorocarbon (CFC) emissions due to air conditioning [31,37,40,45,60,92,[162], [163], [164], [165], [166]], and integrating user involvement [167].

Other examples of decarbonizing the building sector include eco-houses (low-carbon or zero-carbon houses/buildings) [158,[168], [169], [170]], sustainable design principles [171], high-albedo (reflective) roofs [172], and bio-inspired building design [173]. Mitigation efforts using existing infrastructure could include building design principles and retrofitting, and improvement of public transport systems [92,102,131,162,[174], [175], [176]]. Improving the public transport system assists in decarbonizing the built environment by improving accessibility and transport options [121].

Growing global urbanization provides an opportunity for climate mitigation through urban planning, design, governance, and provides an opportunity of achieving a high quality of life with lower emissions [127,154]. Most urban GHG emissions involve energy production, transportation systems, and building heating/cooling systems [92]. Existing, new, and future urban infrastructure can be used to create urban solutions to climate change which address urban GHG emissions. Some limitations and challenges exist, however, in decarbonizing the built environment. For example, the rebound effect due to increased energy efficiency, economic investment in low-carbon building components will need to be substantially increased, and the built environment will also need to be adapted to the effects of climate change which requires effective engineering and planning [177].

#### 3.2.6 Reduction in and management of deforestation

Deforestation and forest degradation releases stored carbon, causes biodiversity loss, affects the remaining forest's ability to absorb carbon from the atmosphere and negatively affects the planet's natural climate stabilizers [[178], [179], [180]]. Deforestation is a significant source of anthropogenic CO₂ emissions (accounting for up to 18% globally) and it also prevents the sequestration of CO₂ from the atmosphere [[178], [179], [180]]. Some scholars propose that in combatting deforestation, livestock and cropland expansion reduction is the best strategy to tackle climate change, biodiversity loss, and infectious diseases [181]. Some important climate change mitigation benefits provided by forests are achieved through storage, sequestration, and substitution [13,49,148,160,[182], [183], [184], [185], [186]].

Forests can also prevent GHG emissions by wood substitution (wood is used instead of fossil fuel-intensive products). Woody biomass can be used for energy, cellulosic ethanol, and avoids land use change [49,184]. A Climate-Smart Forest Economy (CSFE) reinforces these benefits [148]. In a CSFE the aim is to restore, reduce, and safeguard the interests of small-scale growers and forest-dwelling communities [148]. Engaging small-scale forest growers in tropical landscapes can assist with restoration of degraded forest areas and provide renewable, carbon positive wood materials [187]. Existing mechanisms to control deforestation, such as Reducing Emissions from Deforestation and Forest Degradation (REDD+), have been criticized for lack of effective implementation, monitoring, permanence, and leakage (moving deforestation to an unregulated area) [178,179,[187], [188], [189], [190]]. If tropical deforestation is to be stopped and reversed, the global community needs to focus on the link between global beef consumption/trade levels and regional/global change [191,192].

Reforestation has been argued to have the largest maximum mitigation potential in the United States and globally via the reforesting of pasture lands and formerly forested land (creating a large carbon sink) [41,[193], [194], [195]]. Natural forest management of privately owned forests, which includes extending harvest cycles, reduced impact logging, forest fire management, and improved silvicultural practices, has been argued as having the second largest mitigation potential [193,194]. Other potentially valuable strategies include reduced further land conversion (whether for urban expansion, croplands or pasture), carbon sequestration through cover crops, utilizing crops with higher carbon storage potential, improved cropland nutrient management, and carbon crediting schemes (to allow for carbon trading) [131,178,193,194,196]. Scholars argue that deforestation must stop and that regeneration of natural forests for sequestering carbon must be prioritized over all other land use conversions [160,197].

Rapid and widespread afforestation (i.e., creation of new forests) for carbon sequestration has been a strategy employed by countries such as China [182,198]. There are limits to afforestation's effectiveness, however, and researchers have proposed forest management and protection strategies instead (carbon stocks will be increased and limit carbon emissions by avoiding deforestation), and reforestation [13,182,194,199,200]. Afforestation that involves monocultures is also problematic as it may have negative effects on biodiversity [105,201,202] and maladaptation may occur where biodiversity-resilience is key [201,203].

Global tree restoration research has found that an extra 0.9 billion hectares of canopy cover can be restored (excluding existing trees) which would allow for the storage of 205 gigatons of carbon [204]. The potential of Secondary Forests (SFs) on previously cleared agricultural land to sequester CO₂ has been suggested as a low-cost mitigation strategy [191,205,206]. This strategy is particularly useful in regions where deforestation has been greatest, such as the Amazon. Such research has shown that there are no significant differences between SFs and lifetime forests in CO₂ accumulation [205]. For SFs to be effective long-term carbon sinks, however, tree diversity is needed [203,207,208]. Researchers point out that although the global tree restoration efforts can significantly help meet the 1.5 °C warming limit by 2050, climate change could adversely affect tree coverage (a loss of 223 million hectares by 2050), particularly in the tropics [204,209,210].

Agriculture and its continued growth are directly linked with deforestation and the forest degradation [179,197]; in particular, the clearing of forests for pasture is the dominant force behind deforestation [179]. For example, in South America, 71.2% of deforestation is driven by animal agriculture (pasture) [179]. Large agribusinesses intended for the international market have been identified as fundamental drivers of pasture expansion [179]. Further, Brazilian Amazon deforestation contributes 2% to global GHG emissions, and this is a direct result of land clearing for cattle ranching [211]. The conflict between the expansion of animal agriculture and the protection of forests is a major challenge that needs to be addressed through effective policy-making, and improvement of national forest monitoring systems in order to implement more effective forest protection strategies [179]. Understanding the main drivers of deforestation can help countries develop specific policies aimed at mitigating the impact of deforestation and carbon emissions [179].

#### 3.2.7 Nature-based solutions

The concept of Nature-Based Solutions (NBS) addresses climate change through actions aimed at the restoration, protection and sustainable management of natural and modified ecosystems (ocean and terrestrial) [195,209,[212], [213], [214], [215], [216], [217]], protection of biodiversity [216,[218], [219], [220]], and increasing the capacity and capture rate of carbon sinks (both natural and artificial) [62,221,222]. NBS include ecosystem stewardship activities referred to as Natural Climate Solutions (NCS) to increase carbon storage or decrease GHG emissions [186,190,195,214,216,223,224]. NBS aim to resolve the dichotomy between economic growth and sustainability/climate change mitigation [225]. Evidence-based stewardship, restoration, and ecosystem management are of paramount importance to NBS and NCS [214]. NBS address both climate mitigation and adaptation, and at lower cost than many engineered solutions [201,216]. Importantly, NBS have the potential to provide 30–40% of the CO₂ mitigation required by 2030 in order to keep warming under 2 °C compared to pre-industrial average temperatures [203]. Some studies recommend that NBS and NCS be combined with efforts to decarbonize the energy and industry sectors [193,226].

Besides protecting biodiversity, coastal wetlands, and temperate forest protection and management [31,36,60,193,219,227], other NBS include stimulating algae growth on the ocean's surface to capture carbon from the atmosphere [228], and protection of land from erosion (by regeneration of vegetation) [36]. It has been pointed out, however, that climate change itself can have an impact on NBS undermining their performance [225,229]. This impact can be overcome by testing the climate resilience of various NBS with a combination of systems analysis and backcasting (planning methodology) [209,225,229].

NCS are aimed at protecting and restoring forests, mangroves, wetlands, reef restoration, peatlands, tidal marshes, seagrass meadows, grasslands and agricultural ecosystems that can help mitigate climate change [188,190,193,194,214,223,[230], [231], [232], [233]], and thus enhance biodiversity and increase carbon stocks [105,183,184,198,234]. For example, peatlands constitute the largest terrestrial carbon stock, hence peatland restoration's potential as a promising strategy for GHG mitigation [195,235,236]. NCS are regarded as having low technological demand and being cost-effective [190,194,227]. Scholars point out that for NCS to be effective long-term and to protect biodiversity, there needs to be an integrated approach whereby the focus of NCS is to create functional and diverse habitats [224].

As part of climate change mitigation, biodiversity protection should involve conservation and natural resource management [203,219,227,232,237]. Ecosystem functioning can be strengthened by increasing species richness and enlarging natural habitats [227,237] through conservation of tree diversity [207]. Scholars argue that climate change mitigation policies and biodiversity protection policies need to be combined to be effective [238]. Also, higher targets of environmental protection need to be set such as 30% of ocean and terrestrial protection, as opposed to the present 10 and 17% respectively [238].

Projections estimate that adoption of terrestrial-based NCS could allow one-third of global progress toward the Paris Climate Agreement's emission reduction targets to be made by 2030 [190]. Unfortunately, despite the promising potential of NCS, they receive very little funding [194]. This may be due to the perception of NCS as competing with agricultural land use, and indeed this may be the case, as many proponents of NCS also advocate for a reduction in meat (primarily beef) consumption [194,239]. Future food demand and cropland expansion may result in mitigation potential loss of NCS, particularly in the tropics where NCS have the strongest mitigation potential [239].

The literature shows examples of NCS being considered not just in the terrestrial context, but also for the world's oceans. Ocean-based solutions for carbon reduction encompass protection and restoration of coastal ecosystems and seafloor protection [50,240], seabed geological carbon storage [241], ocean energy (wind, wave, tidal energies) [50], marine transportation (low carbon) [231], fishery management (seaweed aquaculture and carbon reduction of fishing vessels) [242], and alkalinization and albedo enhancement [230]. Authors also point out that ocean-based carbon mitigation has the added benefit of not requiring land that would otherwise be used for residential or arable food purposes [50]. Protected area systems in general (both land and ocean-based) can offer climate mitigation potential by optimizing natural solutions by way of storage and capture of carbon [188,243]. This can only be achieved by effective management of protected areas and needs to be on both local and national levels, potentially including several actions such as: improving management to avoid threats (e.g., illegal logging, agricultural encroachment), expanding and increasing protected areas and buffer zones [188,208,243].

Studies examining the use of bioethanol grown in Bioenergy Buffer Strips (BBS) have produced some promising results in reducing GHG emissions, acting as terrestrial sinks and avoiding other negative environmental impacts [244]. Several researchers have proposed that BBS can be utilized not only as buffer zones but also as a NBS for the production of advanced bio-fuels [244]. Agroforestry is a promising solution within the agricultural sector that would enhance soil organic carbon pools, achieve more carbon sequestration on land, and help conserve biodiversity [185,196,[245], [246], [247], [248], [249], [250], [251], [252], [253], [254], [255]]. Agroforestry involves integrating food crops with tree crops and livestock on the same land [247], and alley cropping (planting trees along with crops) [193]. Agroforestry and BBS are examples of the usefulness of NBS in resolving the dichotomy between economic growth and climate change mitigation.

The farming of seaweed has been suggested as a strategy for a reduction in GHG emissions and sequestration including the use of seaweed in animal feed, fertilizers, and biofuels [242,256,257]. Seaweed forests represent an important but often overlooked carbon sink [242,257]. Intensive seaweed cultivation can help achieve two simultaneous goals: bioenergy production and carbon capture/storage [242]. Seaweed forest expansion (e.g., in the Arctic), reforestation, and large-scale seaweed farming can all help towards carbon capture and sequestration [257]. As with many NBS, seaweed can also be negatively impacted by climate change.

The increasing urban population presents an urgent need for NBS in urban environments. The world population is expected to reach 9.7 billion by 2050 (7 billion of that being urban) [[258], [259], [260]]. Green or blue (if aquatic ecosystems are concerned) infrastructure, such as increasing ‘green spaces’, has been proposed as a mitigation and adaptation strategy for climate change [92,102,[261], [262], [263], [264], [265], [266]]. Urban tree planting and urban forests have also been proposed as climate mitigation strategies [102,[267], [268], [269], [270], [271], [272]]. Urban tree planting, however, was found to be less effective at offsetting carbon emissions than cable cars and landfill gas management [267]. Other examples of green infrastructure include green roofs, green facades (green walls) [102,261], urban agriculture systems [262], downspout disconnection, bioswales, permeable pavements [273], green parking and green spaces [261,274], and tree-based intercropping systems [264,266,275]. For these strategies to be effective, however, mainstream implementation and effective management is required [261,271,274] along with cohesive urban forest and climate change policies [268], retention and protection [269], and major investments in retrofitting existing structures or establishing new cityscapes [265].

Humans need to be "rewilding" cities by adopting a holistic approach whereby we take it upon ourselves to conserve, maintain and re-establish the habitats we share with both flora and fauna [102,273]. Cities can attempt to reach these goals through legislation, issuing binding provisions, public education, policy incentives, and public participation [102,263]. Although NBS cannot be a panacea for all climate change issues, the combination of NBS with technology, culture and behavior shifts would be an effective way forward [265]. We can work with nature to be our innovation for the future and our salvation [102].

#### 3.2.8 Litigation, behavioral change, and transformative action

Global conventions on climate change such as the Intergovernmental Panel on Climate Change (IPCC), United Nations Framework Convention on Climate Change (UNFCCC), Kyoto Protocol, Paris Agreement, the European Green Deal, the most recent COP15, and COP26, are non-binding and have being criticized for lack of substantial and fast action [276]. To achieve positive climate outcomes, climate change litigation [276], protests, and civil disobedience have been suggested [277]. Climate change litigation can be used as a catalyst for government action, changes in corporate behavior and public opinion, and creates a precedent for future litigation action [276].

Policy design needs to view humans as endogenous agents [278], and deliver a health-centered approach to climate science [279,280]. Highlighting health co-benefits may be a more effective motivator for behavior change for individuals who are skeptical of anthropogenic climate change [280]. For example, policies targeting reduced red meat consumption to improve cardiovascular health, will have the added benefit of reducing GHG emissions [39,278]. Further, behavioral change should complement technological change [281]. Research shows that even modest behavioral change can result in a 6–16% reduction in emissions per capita [281]. Behavioral changes involve a reduction in meat consumption (or a more fundamentally plant-based diet), less food waste, carpooling, and waste recycling [41,[281], [282], [283], [284]]. Other behavioral changes include fewer children, adopting a car-free lifestyle, avoiding airplane travel [283,285], and human enhancement [106,[286], [287], [288], [289]]. Policymakers should incorporate climate-friendly behavior goals into education and awareness campaigns with an emphasis on multiple health co-benefits (such as human and animal health and wellbeing), and ethical imperatives [281,285,290].

Transformative action is required in the form of fundamental institutional and system changes which involves robust solutions-oriented knowledge for transformation [291,292]. For example, divestment initiatives whereby institutions (governmental, corporate, private) invest in zero-carbon climate solutions instead of fossil fuel industries [293], and provide tax breaks to ‘green’ companies [285]. Instead of a focus on profitability or eco-efficiency, transformative action involves sufficiency (setting limits to consumption) and environmental sustainability [291]. A transformation in dominant normative thinking (individual and collective) needs to occur whereby a long-term and global systems perspective is adopted including the removal of ‘dualism’ between human and nonhuman ecosystems, and the adoption of an interconnected and holistic worldview [291,294]. The issue of climate change can no longer be solved by a traditional linear problem-solving model [294]. Moreover, to effectively encourage individuals to reduce their carbon footprint, climate change messages must be coupled with solutions [295], and an appeal to strong drivers of collectivism such as ‘patriotism’ [296]. Solely focusing on GHG emission reduction does not allow for long-lasting transformative change, where the crisis of global warming is a symptom of unsustainability [291].

#### 3.2.9 Geoengineering

Geoengineering, also referred to as climate engineering, includes a range of major technological innovations designed to mitigate climate change by deflecting solar radiation or increasing the reflective capacity of the Earth's atmosphere to alter the Earth's radiative balance [[297], [298], [299], [300]]. Geoengineering is considered inexpensive, technologically feasible [300], and argued to be the most promising method for rapidly cooling the planet as an emergency method [299,301]. Criticisms include the inability to change regional climate to desirable levels, ozone depletion, detrimental effects on plants, acid depositions, ocean acidification, and availability of solar radiation for solar power systems [297,299,301].

Removal of CO₂ and GHG's from the atmosphere and oceans [297,301], such as ocean carbon sequestration and biochar production [298] have also been suggested. Biochar has been suggested for use in soil for carbon sequestration and to achieve negative net emissions [193,302]. These methods have been criticized for being slow to provide measurable results, expensive, and extremely labor intensive [298]. Further, some forms of geoengineering could discourage efforts to reduce GHG emissions, instead relying on making more clouds or injecting more aerosols to counteract the increasing absorption of radiation by the GHGs.

Much like carbon capture and storage solutions, geoengineering technologies are post-emission solutions [297], and thus may do little to actually curb GHG emissions and alter policies and consumption behavior. Geoengineering may in fact encourage complacency and reduce motivation for individual action such as using ‘climate-friendly’ modes of transport, reducing air travel, reducing meat consumption, and reducing electricity use [128]. Thus, while some geoengineering techniques may become viable in the future, significant GHG emissions reductions are still where most effort needs to be applied [298].

#### 3.2.10 Biotechnology

In climate mitigation, only using organisms and ecosystems that evolved naturally has been challenged by the notion of terraforming with synthetic life [212,303]. This concept refers to the engineering and release of synthetic microbial organisms that can reduce greenhouse gases and help improve carbon sequestration [303,304]. Examples of biotechnology include using enzymes and micro-organisms to make bio-based products (e.g., in food, chemicals, bioenergy), genetically modified crops, soil carbon sequestration, and reduced fertilizer use [10,60,305]. For example, a shift from chemical fertilizers and pesticides to biofertilizers and biopesticides is theorized to decrease GHG emissions and pollution [249]. Further, artificial sequestration in the form of terrestrial, non-biological sinks (e.g., deep coal beds, depleted oil, and gas fields) [222,228], deep oceanic non-biological sinks [221,222,228], and mineral carbonation [62] have been suggested. Unfortunately, some of these processes are extremely expensive and energy-intensive [62]. Moreover, criticisms of such novel ideas include potential negative impacts on biodiversity and ecosystems, and the difficulty in regulating the release of genetically engineered organisms [212].

Bio-engineering trees into “super carbon absorbing trees” has been proposed by bio-technology companies [306]. Critics of super carbon absorbing trees, however, argue that they are inefficient in carbon sequestration and raise bio-diversity concerns of large mono-culture plantations which are susceptible to climate change impacts [306]. Much like super carbon trees, many biotechnology solutions to climate change are under ongoing experimentation. Based on scientific evidence so far, it is unclear whether some of these biotechnology solutions produce actual benefits to mitigating climate change or only offer the ‘illusion’ of doing so, and thus may increase the carbon cost of implementing these solutions [212,306].

#### 3.2.11 Food systems

What we eat has far-reaching consequences for humans, nonhumans, ecosystems, and the planet. A recent study utilizing the Emissions Database for Global Atmospheric Research (EDGAR-FOOD) concluded that when land use change, retail and consumption, fuel and transport, packaging, waste, and industrial processes are taken into account, food systems and agriculture account for approximately 34% of all GHG emissions [7,307]. Others have previously reported that food systems contribute 26–30% of total GHG emissions [12,[308], [309], [310]], or a third of total anthropogenic GHG emissions [197]. More conservative estimates have previously reported food production for humans as amounting to 10–12% of GHG emissions [311,312], but these estimates did not provide as comprehensive of an accounting for human food systems as did the EDGAR-FOOD database. The livestock sector accounts for approximately four-fifths of agriculture's GHG emissions [313,314], with the EDGAR-FOOD database estimating this contribution to be at 71%, which includes land use/land use change activities [307]. The remaining contribution (around 29%) derives from supply chain activities, such as all aspects of processing, packaging, transport, and retail [307]. Reducing GHG emissions from the global food sector is essential for preventing global average temperatures increasing beyond 1.5 °C [12]. Some food systems solutions include composting, conservation agriculture (e.g., crop rotation) [31,60,315], yield increase and reduced crop waste [316], changes in food production [317,318], and adopting a reduced red meat/vegetarian/vegan diet [6,40,[319], [320], [321], [322]]. GHG emissions from the food system alone are enough to exceed the 1.5 °C limit [12] and if nothing is done, the global food system's environmental effects could increase by 50–92%, reaching levels which are beyond what is sustainable for humanity [321].

Animal agriculture is the main contributor of methane and nitrous oxide (CH₄, N₂O), and contributes between 52 and 84% of global emissions of these gases [6,[323], [324], [325]]. The major challenge for animal agriculture, particularly the ruminant sector, is a reduction of methane and nitrous oxide emissions. Strategies proposed include ruminant vaccines, genetic selection, dietary manipulation and chemical additives [326], better breeding practices [327], improved fertility [328], improvements in pasture management/regenerative grazing [61,211,329], fertilizer and irrigation [324], optimized lifetime efficiency of dairy cattle and production [327,330], reduction in energy and water use [39], and manure management [328,331,332]. As with many other animal agriculture strategies, however, these approaches assume a ‘business-*as*-usual’ model and fail to address the inherently climate-damaging effects of an increasing demand for meat and dairy. Some literature reviews have concluded that a combination of animal feeding and housing practices could help reduce emissions [333], without considering dietary shifts or adjustments. Researchers have found that a 30% reduction in livestock production and improvements in agricultural technology would meet GHG emission reduction targets by 2030 for countries such as the United Kingdom [314]. The authors do point out, however, that such moves would encounter substantial resistance from various interest groups [314].

Technological innovations in agriculture, while having the capacity to reduce non-CO₂ GHG emissions, are less effective than changes in consumption [334]. There must be a concomitant shift towards adoption of a low-carbon diet, particularly in high-consuming, high-income countries [314]. Mitigation of climate change requires a close look at the animal agriculture industry and immediate significant changes in patterns of consumption, production, and regulation [325]. For example, a reduction in production and consumption of low-efficiency food products most notably, ruminant meat and milk products will have significant impact [192,335,336]. Furthermore, increasing production and consumption of energy efficient food products, using residual carbon for energy production, and optimized land use will help greatly with climate change mitigation [335]. Some agricultural land made available as a result of changes in food production could also be used to sequester carbon and grow lignocellulosic crops to substitute coal in power generation [337,338].

Proponents of livestock farming, in particular beef and dairy, have argued that it can contribute to carbon capture, however, this argument ignores the fact that to achieve this, the number of animals per hectare has to be much lower than would allow for present consumption [336]. Some authors have proposed strategies to specifically curb beef production/consumption. For example, putting a stop to beef subsidies and beef consumption promotion (in both high-income countries and new consumer societies), and a tax on meat production and consumption [337,339]. The taxes collected could then be used to further develop technological innovations to reduce GHG in animal agriculture and aid farmers to switch from animal agriculture to other non-animal agriculture [339]. Other suggestions for improving future agricultural land use to mitigate climate change include controlling soy bean production for animal feed and grazing (to stop deforestation and savanna conversion) [191], protecting/restoring forests on grazing land and allocation of resources to less environmentally damaging land uses [318,340], cultivating organic soils, enhancing management of crop and grazing land [328,341], and restoring degraded lands [323].

Emissions from ruminants such as cattle and sheep far outweigh those from fruits and vegetables, unless they are transported by plane or produced in flooded rice fields [61,342]. The lowest emission plant-based foods are legumes, fresh vegetables, and cereals [342]. Some scholars are now pointing out that a transition to a more plant-based diet (and excluding animals with enteric fermentation) must become part of the climate mitigation response [192,283,339,342,343]. Research has found that a switch to diets containing less animal-source foods could cut GHG emissions from food production by up to 40% [284]. This research, however, falls short of suggesting a switch to a plant-based diet as researchers deem it unacceptable to the general public [284]. Instead, scholars suggest a combination of waste reduction [282], less meat/dairy in the average diet [192,284,321,339,344,345], technological improvements [331,346], genetically-modified crops [305], climate-smart agriculture [[347], [348], [349], [350]], and increased production efficiency as a way to achieve the required reduction in food GHG emissions [192,323,330,341].

Scholars propose that the most climate efficient diet is one rich in legumes, cereals, and in some cases fish [256,342]. Specific to fish production methods, small pelagic fisheries and mollusk aquaculture were found to have the lowest impact on GHG emissions, while catfish aquaculture had the highest [308]. In terms of fishing methods, seafood caught with trawling has three times the emissions of non-trawling methods [322]. Scholars acknowledge, however, that most fish stocks have been overexploited and industrialized fish farming carries a heavy environmental burden, leaving only plant-based options [308,342]. A plant-based diet has less effect on the ecological footprint [317,351] and has the lowest relative impact in various categories of energy and land use, and GHG emissions [352].

Beef production and dairy are the most unsustainable animal agriculture industries [6,79,342,351,353]. Research has shown that beef is the least efficient protein and has the highest GHG emissions intensity [6,282,308,320,336,342,343,354]. The emissions per gram of protein for beef is almost 250 times that of plant-protein, and even 20 servings of plant foods compared to one serving of beef have lower GHG emissions [79,322]. Even when compared to per unit of protein, plant-based foods hold more benefits over animal-based foods in terms of GHG emissions [336,355,356]. Other sources of protein such as lab-grown meats, micro-algal proteins and insect-based diets enable less polluting protein production [357]. Moreover, if global meat consumption was decreased, global agricultural non-CO₂ GHG emissions would also decrease (even compared to 1995 levels) [334]. If no improvements are made, emissions from the animal agriculture industry could double by 2070 [344]. Most research focuses on reducing GHG emissions on the supply-side of the livestock production system with little attention given to the demand-side [358]. A reduction in meat consumption would reduce meat production, in turn, reducing GHG generation [79,317,358]. Moreover, a reduction in meat consumption could be one of the drivers of a gradual global phaseout of industrialized animal agriculture.

#### 3.2.12 Phaseout of industrialized animal agriculture

The livestock sector alone accounts for 25–30% of global GHG emissions [197,307]. Some of the more conservative estimates of animal agriculture's contribution to climate change do not take into account fuel used in agriculture, land use, emissions from other sectors such as buildings and industry, water use, eutrophication, deforestation and biodiversity loss, desertification, and historical land use change [6,197,211,308,325,328,333,336,359]. Scholars estimate that taking into account the historical transformation of land for livestock, animal agriculture accounts for one-third of all anthropogenic CO₂ emissions to date [6,211,358,360]. Livestock's GHG emissions have been estimated at up to 2.5 times higher than that of all global transport combined [192,312,358,361,362]. Animal agriculture also contributes far more to deforestation, ocean acidification, species extinction, and biodiversity loss than plant-based foods [309,319,363]. Around 70% of agricultural land is used to either house or feed animals [309,312,322,343,345,362], 70–85% of the global water footprint is attributable to agriculture [361], and 30% of Earth's surface is directly or indirectly involved in livestock production [312]. Even the lowest-impact animal products have a greater impact on the climate than plant products, with scholars arguing that this alone provides evidence of the importance of drastic dietary change [309]. Moreover, the efficiency of plant-based foods increases (low energy and GHG emissions) as the protein levels in the foods increase, whereas the opposite is true for animal-based foods (higher protein levels mean higher energy and GHG emissions) [336,342].

Eating meat contributes almost four times as much to GHG emissions as a plant-based diet [309]. Animal industries producing meat, eggs, dairy, and aquaculture occupy approximately 83% of the world's farmland, while only providing 18% of our calories and 37% of our protein [309]. A plant-based diet adoption on a global scale has the transformative power to reduce demand for land by 76% (including a 19% reduction in demand for arable land), a 49% reduction in food's GHG emissions, reduced acidification by 50%, a 49% reduction in eutrophication, and a 19% reduction in freshwater withdrawals [309]. Even highly processed plant-based proteins and vegetable milk (made from lentil proteins) have been found to have significantly less environmental impact than meat (mass and protein content taken into account) [356]. A shift away from animal-based diets to plant-based diets is mutually beneficial from both nutritional and environmental perspectives [315,317,336,342,356]. The increased intensification of livestock and growing demand for meat will only compound the negative impact animal agriculture is having on the planet [364].

It is expected that the meat industry will expand its operations by 50–73% by the year 2050 to meet the growing demand for meat of the projected 9 billion humans [365]. If our current eating habits continue and meat consumption doubles by 2050 [362,366], approximately 80% of existing forests and shrubland will need to be converted into land for raising animals for dairy, eggs, and meat [7,367]. This would have a devastating impact on the climate and is unsustainable. An additional 35 million km^2^ of land, roughly the area of Africa and Australia combined, would be required for livestock production if the industrialized world's animal-rich diet is extended globally [6].

There is also a growing adoption of intensive livestock farming methods, referred to as Concentrated Animal Feeding Operations (CAFOs) among both industrialized and less-industrialized nations which will only increase GHG emissions [325,364]. A study proposing a ‘beans for beef’ shift in diet found that substituting plant food for meat could free up 42% of US cropland [368]. The authors concluded that swapping animal-based food with plant-based food would make a significant contribution to climate change mitigation, especially if other mitigation efforts are implemented in sectors such as transportation and energy production [368]. The authors extend their argument for plant-based foods by incorporating meat analogs and propose that alerting consumers to the health benefits of such a dietary shift may increase consumer interest and behavioral change [282,338,368].

If novel foods (such as lab-grown meat, milk, plants, algae, bacteria, fungi) and future foods (insects, spirulina) were to replace animal source foods, animal agriculture's environmental impact would be reduced by 80% (global warming potential, land/water use) [369]. Lab-grown meat requires 99% less land than animal agriculture, 90% less water, and 45% less energy [370]. In addition, facilities for lab-grown meat can be placed in areas that are inhospitable to livestock production and increase food security [370]. Research has found soy-based and insect-based substitutes to have the lowest impact, and lab-grown meat and myco-protein based substitutes to have the highest [361], such as in energy use [371]. The authors point out that as technology for lab-grown meat becomes more efficient, the energy requirements of this meat substitute may be significantly reduced [361].

There is growing consensus among climate change researchers that the window of time during which we can enact meaningful change is much shorter (7–8 years) than previously thought [3,7,11]. Scholars have proposed that an immediate solution to the problem would be to end all animal production, encourage a more sustainable diet, and a switch to a plant-based or vegan diet incorporating low-carbon producing vegetables [6,283,309,316,317,319,336,342,343,345,368]. A vegetarian or vegan diet is 20–55% lower in GHG emissions compared to a typical Western diet [372]. Reducing animal production would have the added benefit of more land available as carbon sinks [316,317,345,352]. Afforestation on freed up land would achieve a GHG emission reduction of 4–37% through sequestration [352].

From an individual action perspective, humans have the capability to significantly lower their impact on climate change by adopting a meat-free (or predominantly meat-free) diet [336,[373], [374], [375]]. The animal agriculture industry works on the principle of supply and demand; by eliminating or reducing demand, production can be reduced [364]. Effective strategies to influence individuals to change their diet have been found to involve the perceived threat to “others” (other people, animals), increasing self-efficacy and response-efficacy beliefs (e.g., linking food and climate, smaller portions, energy conservation), and providing consumers with viable options to replace meat intake [82,373]. With critical consumers (those that are climate change skeptics), authors propose a strategy for meat reduction that involves an emphasis on human health or factory-farming critiques [373].

Previous research has found that a reluctance to advocate for a plant-based diet from governments, politicians, and environmental groups may be due to the belief that dietary changes are harder to “sell” to the general public than changes in energy/transport, and a reluctance to take on powerful lobbying groups in the agricultural sector [319]. Researchers suggest that mass media can have an important role to play in encouraging consumers to consider switching to low-carbon or plant-based diets [319]. Although this type of approach has been criticized for shifting the focus from corporations/governments to individuals, it is the single most effective individual response (plant-based/vegan) for reducing one's carbon footprint and is still the least mentioned strategy in mass media [319].

Even if no other emissions are reduced, a phaseout of animal agriculture would provide 52% of the necessary net emission reduction to limit global warming to 2 °C by 2100 [6]. In terms of specific animal products, 47% of the benefits of a phaseout of animal agriculture is accounted for by beef alone, while cow milk accounted for 24% [6]. Crucially, the complete phaseout of animals in the food industry would produce substantially reduced emissions that even a complete replacement of fossil fuels could not achieve on its own (to limit global warming to 1.5 °C) [6]. The scholars argue that along with a transition to renewable energy, a global change in diet is a powerful mitigation tool, and instead of a selective focus on any one strategy, political, social, and economic barriers must be overcome to implement these mitigation strategies successfully [6].

If a global dietary change occurs (to a plant-based diet), carbon capture and storage technology may not be required [360]. Further, a dietary change has the potential to mitigate 49–70% of annual ‘business-*as*-usual’ food system emissions (non-CO₂ emissions) [360]. Overall, shifts in global food production to plant-based diets by 2050 could result in a 66% chance of limiting global warming to 1.5 °C [360]. Although global dietary shifts would be economically disruptive and carry socio-cultural costs, these potentially negative effects must be weighed against the future costs of unabated agricultural emissions [360]. High-income and high-emitting countries could financially support restoration efforts of high-carbon forests, food security, livelihoods, and agricultural productivity in LMICs [360]. Further, the warming potential of the planet's atmosphere would be frozen for 30 years if a gradual total phaseout of animal agriculture occurs within 15 years [6], and 68% of current anthropogenic CO₂ emissions could be offset [6]. Scholars acknowledge that animal agriculture supports the livelihoods of as many as 1 billion people worldwide, however, with minor nutritional adjustments, existing crops could replace animal-source foods in terms of protein, fat, and calories, and greatly reduce the carbon footprint of food [6,376].

#### 3.2.13 Climate change metrics and regulatory reporting requirements

Although carbon related disclosures remain voluntary for the most part and no universal standard of reporting exists, carbon footprint disclosure is a growing practice [377,378]. Research has shown a positive relationship between environmental disclosure and financial performance [[379], [380], [381]]. In an attempt to standardize carbon disclosure, organizations such as the Global Reporting Initiative (GRI), Sustainability Accounting Standards Board (SASB), and Carbon Disclosure Project (CDP) have been created [382]. Criticisms of some of these initiatives include that they are voluntary, companies do not need to provide full disclosure and there is no auditing process or other way to verify the accuracy of information provided by companies [377]. Some initiatives (such as the CDP) are funded externally by a variety of organizations and while it maintains the claim of independence, there have been criticisms relating to its legitimacy and overt bias [377].

Mandatory initiatives have also emerged. For example, facilities emitting at least 25,000 metrics tons of CO₂ equivalents per year are required to disclose their emissions in the United States [383,384]. Authors raise several important considerations regarding carbon disclosure and regulatory requirements such as mandatory versus voluntary reporting [383]. Some research has shown that climate change-related disclosure is negatively affected by co-existing regulation and mandates [384] while other data have suggested a positive impact on climate change disclosure [381].

Environmental, Social and Governance measures (ESGs) are examples of metrics used to describe the sustainability, transparency, and performance of a company, where the ‘E’ represents a company's contribution to climate change. Metrics underlying ESG measures remain diverse particularly regarding the choice of indicators which measure ESGs. There is currently no standard definition of what constitutes ESGs, and what aggregation methodologies and weights should be applied to each indicator [382,385]. A vast number of ‘data vendors’ provide products which rank companies and offer companies ESG overall rating scores. Transparency is lacking regarding which indicators and methodologies are used and organizations sometimes use data from ESG data vendors to create their own rankings [382]. Collective criticisms include a lack of transparency, commensurability, reliability, and validity [382,385,386]. These shortcomings make it difficult to evaluate true ESG performance of companies and companies may be disincentivized to improve their ESG performance due to unreliable and inconsistent ESG ratings [385]. Overall, the effectiveness of climate change metrics is hindered by a lack of standardization in measurement. Future use of climate change metrics to help mitigate climate change can only be effective if measures used are rigorously defined and standardized.

## 4 Discussion

Our planet is in a state of emergency and we only have a short window of time (7–8 years) to enact meaningful change to prevent an eventual global climate crisis that will impact each and every one of the Earth's inhabitants [3,7,11]. Our consumption habits, dietary choices, and economic/industrial priorities, are threatening the existence of all life forms on this planet, including humans [7]. GHG emissions need to be reduced by 45% to limit global warming at 1.5 °C above pre-industrial levels, if we want to avoid irreversible global climate change [2,3,7,11]. To achieve this monumental task in such a short space of time, human activities cannot continue on a ‘business-*as*-usual’ basis.

This literature review provides a systematic overview of climate change mitigation solutions identified in the last 20 years (2002–2022) and proposed by researchers in peer-reviewed academic publications. Solutions such as a switch to renewable energies and fuel, changes in the built environment, nature-based solutions, improved agricultural technology, and many others have been given significant weight among the scientific community. Yet what has become obvious is the omission or downplaying of a major solution that has the potential to offset our total GHG emissions by as much as 68%: the global shift to a more fundamentally plant-based diet and phaseout of industrialized animal agriculture [6]. Moreover, the warming potential of the atmosphere would reduce significantly if the gradual phaseout of industrialized animal agriculture is achieved within the next 15 years, which can give scientists a chance to develop and advance new and existing technologies [6]. We have chosen to focus on a gradual phaseout of industrialized animal agriculture (factory farming) because the vast majority of animal agriculture-related climate change, as well as human, animal and environmental health risks derive from factory farming [387,388].

There are several commonalities among the proposed climate change solutions. The role of nature, as a relatively low-cost solution, in addressing climate change has received much attention. Another major commonality relates to the resources we use such as energy/fuel, transport, buildings, and infrastructure. Researchers also identify steps that individuals can take to reduce their environmental footprint such as waste reduction, changing of consumption patterns, lifestyle changes regarding the use of vehicles and food choices, and a shift in mindset (such as reducing consumerism). On a global scale, societal transformation can be achieved through education and global cooperation that transcends political, social, and economic divisions, and demonstrated by global initiatives such as One Health [[389], [390], [391], [392]]. The shift in collective mindset can be assisted by educational initiatives such as the 1 HOPE (One Health for One Planet Education) global initiative [393]. Such initiatives can modify the collective mindset from being heavily anthropocentric and exploitative, and should encompass all spheres of society, such as academia, governmental, corporate, and the general public [7,393]. Global cooperation is required to achieve this and a recognition among the world community that the survival of all species is dependent on a healthy planet [4,393].

Many researchers propose plans of action that are too slow and too late – aiming for global change by 2050, when solutions and actions are needed in the next 7–8 years. Moreover, many of the solutions that have been proposed attempt to introduce new technologies to allow humanity to continue behaving and consuming in much the same ways. It is clear, that along with technological innovations, humans must change their behavior and consumption habits, along with our mindset. Collectively we need to recognize the sanctity of life and strive for the well-being of the planet and all its inhabitants [87]. We need to shift away from viewing the planet and its resources as limitless and strive for shared prosperity and global stability [87].

Some promising solutions to achieve change in a shorter time span include the removal of tax subsidies in the transportation and food sectors (particularly in the industrialized animal agriculture industry) [55,136,191]. Essentially, a removal of tax subsidies in industries deemed most harmful to the climate such as the beef and dairy industries [191]. Further, decarbonizing in emerging economies and alternative microbial biofuels also seem promising [10,45,53,73,121,125,129,134,140]. The growing urbanization trend provides the opportunity for climate mitigation through urban planning and design, including green infrastructure, with the “rewilding” of cities being a promising and important solution. Notably, nature-based solutions could provide 30–40% of the CO₂ mitigation required by 2030 to cap warming at under 2 °C and can be utilized alongside other major solutions such as the gradual global phaseout of industrialized animal agriculture [203]. We must recognize that solely focusing on GHG emission reduction does not allow for long-lasting transformative change, given that the crisis of global warming is a symptom of unsustainability [291]. Long-lasting transformative change can only be achieved by changing individual, corporate, nation-wide, and multi-national behaviors and mindsets.

The switch to fully renewable energies will be insufficient for us to achieve our needed climate change goals, we must change what we eat, and this must be done globally. We recognize that there is no one solution to climate change and that a combination of solutions may be most effective. Arguably, two of the most impactful solutions are fossil fuel and industrialized animal agriculture phaseouts. Given the concentrated focus on fossil fuels, researchers and policymakers also need to investigate how to achieve a global agricultural and dietary shift and incorporate recommendations into policy. Policy decisions can drive the reduction in consumption of animal-source foods and thus, significantly reduce the environmental costs of food production [211,363,394]. Importantly, the phaseout of industrialized animal agriculture can buy us time to develop technologies for a full fossil fuel phaseout and effective long-term carbon storage. The gradual global phaseout of industrialized animal agriculture will also assist in ending deforestation (itself a major and increasing driver of climate change) and promote reforestation and afforestation which are crucial to our ultimate success in reversing climate change.

Although some scholars show and discuss a clear link between animal agriculture and climate change, the reduction and phaseout of animal agriculture (particularly industrialized animal agriculture which comprises approximately 70% of the world's food animal production and increasingly dominates this arena) is almost entirely overlooked including in many ‘comprehensive’ climate change solutions [179,325,344,387,388]. A few researchers propose the phaseout of animal agriculture [6], with some arguing that this is unachievable because it is either unacceptable to the general public, involves powerful and influential interest groups, or too costly to implement [284,319,395]. However, as research has shown, the general public are against many of the cruel and inhumane practices used in industrial animal agriculture and the effects that it has on human health, as well as the health of communities and the environment [[396], [397], [398], [399]].

We propose, along with other researchers, an increased emphasis on the health benefits of adopting a plant-based diet to improve its public acceptance [400,401]. For example, a plant-based diet has the potential to reverse a host of cardiometabolic chronic diseases such as coronary atherosclerosis and diabetes [[401], [402], [403], [404], [405]]. A plant-based diet reduces the relative risk of cancer by 15% and offers neuroprotection against cognitive aging [401,406,407], and following a predominantly plant-based diet reduces risk of all-cause mortality compared to diets containing animal-based foods [401,408]. Furthermore, for members of the public who are highly skeptical of transitioning to a more plant-based diet, lab-grown meat could prove to be a particularly important alternative. Lab-grown products are the same as regular animal products in nutritional composition, texture, and taste, without all of the toxins, infectious agents and environmentally damaging elements that come with farmed meat.

Although some assert that phasing out of animal-based agriculture is unfeasible, due to what is often argued as its role in feeding the whole world in a “cost-effective and sustainable manner”, we contend that the animal agriculture industry or industrialized farming is grossly unsustainable [409]. By eliminating the use of animals for food, we could feed far more people and use far fewer resources. As an example, for every 100 calories of cereals used to feed food animals, only 17–30 calories are delivered as meat to humans [410,411]. A staggering 77% of agricultural land is used by animal agriculture, but produces only 18% and 37% of global calories and protein, respectively [412]. Externalized costs of animal agriculture produce the illusion that animal-based products cost less, particularly those produced on factory farms. Externalized costs are passed on to others outside the industry, including costs related to government subsidies and adverse impacts upon climate change; pollution (soil, water, and air); and various aspects of human and animal health [409]. The products of factory farms are artificially cheap, and policies requiring factory farms to pay for the pollution they cause would dramatically change this [413]. The impacts factory farms have on the environment, animal and human health/welfare, and rural communities, are not reflected in the prices of the resulting animal products [413,414]. Plant-based foods have the smallest carbon footprint and adoption of a plant-based diet has the potential to reduce GHG emissions by up to 70% [415,416]. Thus, the industrialized animal agriculture system provides trivial benefits to which viable alternatives exist [414].

The costs associated with the adverse impacts of factory farming are absorbed by taxpayers, societies, governments, future generations, the planet, and amount to trillions of dollars per year globally [409,415,417]. Further, industrialized animal farming prioritizes efficiency and profit maximization over safety and this is reflected in high rates of injury and infection among workers in the industry, who are often marginalized immigrants or people of color [401,418]. Routine administration of antibiotics to accelerate growth and prevent infection, overcrowding of animals, and suboptimal sanitary practices increase the risk of antibiotic resistance, spread of diseases and zoonotic pandemics [401,409,[418], [419], [420], [421]]. The question that confronts humanity is whether we have learned the lessons from the COVID-19 pandemic and its zoonotic origins to prevent future pandemics [422].

Our heavy reliance on animal products, as an inefficient way to feed the global population, increases the risk of human starvation, zoonoses/pandemics, antibiotic-resistant infections, and a global climate change crisis [409]. The risks of many noncommunicable diseases and disorders are also increased such as diabetes mellitus, cardiovascular disease, ischemic stroke, obesity, Alzheimer's disease, and different forms of cancer [401]. Given the Food and Agriculture Organization of the United Nations (FAO) estimate that to meet a growing demand for meat, the production of animal products would have to double by 2050, it is impossible to meet this demand without converting most of the remaining forests to animal agricultural land – unless a dietary shift occurs towards a plant-based diet [362,409]. A recent University of Oxford study found that a transition toward more plant-based diets could reduce food-related GHG's by 29–70% by 2050, and avoid 5.1 million human deaths per year [415]. Whereas a shift to totally plant-based diets could result in even greater GHG benefits and avoid 8.1 million deaths per year [415]. In economic terms, the economic benefit of a shift to plant-based diets was calculated to be $31 trillion per year (13% of global GDP) by 2050 [409,415]. We recognize that adoption of a plant-based diet and global phaseout of industrialized animal agriculture will need to be gradual, yet certain actions can be taken quickly to start a change in this direction.

### 4.1 Three new strategic approaches

The authors of this article recommend three strategic approaches. **Firstly**, we propose the gradual shift to a plant-based diet and global phaseout of industrialized animal agriculture. Animal-based meat, dairy, and egg products can be increasingly replaced by plant-based and lab-grown products. To achieve this, further developments and investment are required in technologies which allow for the creation of cheaper, more widely available, and tasty meat, dairy and egg alternatives [423]. The potential of plant-based and lab-grown meat alternatives is increasingly obvious both to entrepreneurial ventures and also to major meat producers [424]. Additional investment is needed in plant-based agriculture which, as previously mentioned, would feed more humans and use less resources while also preserving ecosystems [421]. As previous research has shown, animal agriculture is not necessary (nor sustainable) to feed the growing global population and with minor nutritional adjustments, existing crops could replace the fat, calories, and protein of animal-source foods, with a greatly reduced carbon footprint [6,321]. Also, the land freed from animal agriculture could be used to restore natural habitats or grow new crops for food or power generation. Plant-based meat alternatives can be made cheaper through tax cuts and funded health campaigns to reduce animal-based meat, dairy, and egg intake.

Alongside the adoption of a plant-based diet, we recommend the gradual global phaseout of industrialized animal agriculture. Industrialized animal agriculture or factory farming (also referred to as Concentrated Animal Feeding Operations, CAFOs) should be clearly distinguished from other forms of animal agriculture in large-scale informational activities and other strategic initiatives. We define factory farms as “all operations that include industrial livestock production practices in which densely populated groups of animals are confined to cages, barns, or feedlots” [409]. In factory farms, the water, feed, and medical inputs are provided to the animals, and “their excrement collected in ponds (called lagoons) or pits, to be sprayed onto nearby fields” [409,425]. A similar definition of factory farms is used by the United States Environmental Protection Agency which bases its definition upon industrial methods and the potential of factory farms to a be significant contributor to pollutants [409]. Based on these definitions, over 98% of U.S. farmed animals and 70% of farmed animals globally live on factory farms [387,388]. Factory farming poses a major global threat to not only climate change and environmental pollution, but also to human and animal health and the ongoing threat of pandemics [421], and thus should be the primary target. Small scale farms (which do not fit the industrial animal agriculture definition discussed above) have a much smaller carbon footprint and do not compete with the industrialized animal agriculture industry in terms of their impact on health, climate, and environmental pollution [413,414].

Industrialized fish farms also carry a heavy environmental footprint and strategies used to gradually phase out industrialized animal agriculture should also target intensive fishing practices [308]. Thus, our proposal for the global phaseout of industrialized animal agriculture encompasses factory farming of all land and water animals. This clarification is important because most previous research on the impact of animal agriculture has focused on ruminant and red meat. White meat's (such as chicken and fish) climate impact is often undercounted [416]. However, when land use changes, water usage, eutrophication potential, manure and nitrogen generation are factored in, white meat's impact on climate change becomes comparable to that of beef [416]. Also, this impact could become much worse as people reduce red meat intake and substitute it with chicken or fish in order to consume more ‘environmentally friendly’ foods while maintaining animal-source protein intake.

To achieve a gradual global phaseout of industrialized animal agriculture, we propose ending government subsidies for animal-based meat, dairy and eggs and initiating taxes on such products to account for the externalized costs related to climate change and other factors mentioned above. Taxes can be introduced gradually and on a differentiated basis (in line with some previous research). Specifically, a differentiated tax on ruminant meat/milk (as being the most environmentally damaging) followed by a non-differentiated tax on all meat/milk to reduce all animal-based foods consumption [339]. The taxing of ruminant meat, however, may increase consumption of pork and poultry products which are almost entirely factory farmed [426]. Therefore, taxes instead could be applied to all animal products from industrialized farming practices. Removing tax subsidies for industrialized fishing practices has also been suggested [55,136].

Taxes would need to be introduced in high-income countries first. The taxes and money saved from government subsidies could then be used to develop technological innovations in alternative meat/milk products and aid farmers in their transition from animal agriculture to non-animal agriculture. The goal is for lab and plant-based alternatives to become competitive with industrialized meat production. Other actions specific to factory farming could include more stringent legislation on animal welfare standards, divestment in industrialized animal agriculture, legislative limitations on where factory farms can exist, and mass media public information campaigns that more clearly outline the benefits of plant-based nutrition and the present and growing dangers that are inherent in industrialized animal agriculture [409].

Corporate investment in animal agriculture industries is becoming increasingly high-risk because of the increasing negative impact of climate change on these industries. Divestment from companies engaged in industrialized animal farming is critical if we are to achieve less environmentally damaging food sources [426]. Many of the so-called economic benefits of the animal agriculture industry are associated with market distortions in the form of negative externalities, loss of natural capital, credits, and subsidies [427]. As some scholars suggest, industrialized animal farming poses an unacceptable level of risk to global economies [426].

We recognize that a gradual global phaseout of industrialized animal agriculture will impact many rural communities and communities in LMICs whose livelihoods rely on animal farming. Investments on a local and global scale are needed to assist this major change and the onus is on wealthier nations to help drive this change. The global cost to humanity of not acting on industrialized animal agriculture will be much greater than the cost of phasing it out. The failure to take action will ultimately result in a scenario of irreversible climate change with widespread famine, global environmental and agricultural devastation, climate refugees/warfare, flooding, and disease [7].

**Secondly**, to assist with the shift to fundamentally plant-based diets and the global phaseout of industrialized animal agriculture, a shift in global mindset is required. Specifically, we suggest the adoption of an All Life approach in global mindset, the scientific community, government policy/action, corporate behavior and policy. An All Life approach recognizes the profound interconnectedness of all life on our planet, its protection, and shifts away from a human-centric paradigm to an Earth-centric paradigm [7]. An All Life approach emphasizes the protection of the ‘oneness of life’ (us, animals, plants, the entire planet) and emphasizes that our health and the health of our planet are intimately intertwined with the health and wellbeing of all living beings. As a solution to climate change, we must work toward a sustainable scenario for our planet which ultimately includes the adoption of a more fundamentally plant-based diet and the phaseout of industrialized animal agriculture, benefitting humans, non-humans, all ecosystems, and our planet. A change in global mindset can be achieved through education and awareness campaigns [7]. Those involved in the human, animal and environmental protection communities must come together and join forces to forge an All Life protection movement. We are running out of time to alter our current trajectory, and thus to enact meaningful change that will have a profound impact upon the future wellbeing of the planet and all of its inhabitants.

**Thirdly**, regarding ESG measures, the lack of standardization negatively affects the reliability and validity of ESG scores and rankings, affects the trustworthiness and transparency of company disclosures, and disincentivizes companies from improving their scores. Accordingly, we propose an urgent need for the global standardization of ESG or similar measures and the introduction of a regulatory body for verification of such measures, particularly the measure of environmental impacts as they relate directly to the problem of climate change. To ensure the credibility of such a regulatory body, it must be an independent, not-for-profit entity to remove potential vested interests and biases. Such measures will have a fundamental impact upon corporate and governmental performance, accountability and effectiveness while providing important guidance for individual and institutional investors.

### 4.2 Limitations

Only articles written in English were reviewed in this systematic analysis, which may have introduced bias, gaps in understanding of global climate change, and an omission of Indigenous and local knowledge [428]. Articles published prior to 2002 were not included (due to set parameters of systematic review); solutions proposed in these articles may still be relevant today. It was thought, however, that any such solutions were likely to be discussed in the twenty-year period (2002–2022) included in the parameters of the review. Several articles were unavailable through the search engines used. Further, many articles covered the same strategies for climate change mitigation, which made it difficult to present a broad scope of climate change solutions.

## 5 Conclusion

Based on the findings of this literature review, we suggest a new path forward to solving the global climate crisis. We propose an emphasis upon three new strategic approaches [1]: the gradual shift to a plant-based diet and global phaseout of industrialized animal farming [2]; a shift to an All Life approach that recognizes the profound interconnectedness of all life on the planet; and [3] a global standardization of climate change metrics. These strategies will be needed to solve the global climate crisis and will complement the gradual elimination of fossil fuels and the other promising time-sensitive solutions that offer the greatest promise within a 7–8 year window including: the removal of various tax subsidies in industries deemed most harmful to the climate; decarbonizing in emerging economies; alternative microbial biofuels; climate mitigation through urban planning and design; and other Nature-Based Solutions including green infrastructure and “rewilding” of cities. We must recognize that by solely focusing on reducing GHG emissions to limit global warming, we are treating the symptom of the cause, and the cause is major global unsustainability. To achieve long-lasting transformative change, which will benefit current and future generations (and save our planet), we need to change our mindset and behavior as individuals, communities, businesses, governments, and global citizens.

## Data availability statement

Data included in article/supplementary material/referenced in article.

## CRediT authorship contribution statement

**Svetlana V. Feigin:** Conceptualization, Data curation, Formal analysis, Investigation, Methodology, Visualization, Writing - original draft, Writing - review & editing. **David O. Wiebers:** Conceptualization, Data curation, Formal analysis, Investigation, Methodology, Writing - original draft, Writing - review & editing. **George Lueddeke:** Writing - review & editing. **Serge Morand:** Writing - review & editing. **Kelley Lee:** Writing - review & editing. **Andrew Knight:** Writing - review & editing. **Michael Brainin:** Writing - review & editing. **Valery L. Feigin:** Writing - review & editing. **Amanda Whitfort:** Writing - review & editing. **James Marcum:** Writing - review & editing. **Todd K. Shackelford:** Writing - review & editing. **Lee F. Skerratt:** Writing - review & editing. **Andrea S. Winkler:** Writing - review & editing.

## Declaration of competing interest

The authors declare that they have no known competing financial interests or personal relationships that could have appeared to influence the work reported in this paper.
